# Technical Accuracy of Dental Laboratories in the Quality and Shade Matching of Porcelain Fused to Metal Crowns: An In Vitro Study

**DOI:** 10.3390/ijerph18052722

**Published:** 2021-03-08

**Authors:** Mohammed. S. Bin-Shuwaish, Yasser F. AlFawaz, Hamad A. AlGamaiah, Abdulaziz S. AlSani, Ibrahim B. Abobakr, Khaled M. Alzahrani, Basil Almutairi, Esraa A. Attar, Fahim Vohra, Tariq Abduljabbar

**Affiliations:** 1 Department of Restorative Dental Sciences, College of Dentistry, King Saud University, 60169, Riyadh 11545, Saudi Arabia; malshowaish@ksu.edu.sa (M.S.B.-S.); yalfawaz@ksu.edu.sa (Y.F.A.); algamaiah@gmail.com (H.A.A.); ibabakr@gmail.com (I.B.A.); balmutiri@ksu.edu.sa (B.A.); 2 Graduate Restorative, School of dentistry, University of Michigan, Ann Arbor, MI 48109, USA; assani@gmail.com; 3 Department of Prosthetic Dental Sciences, College of Dentistry, Prince Sattam Bin AbdulAziz University, Alkharj 11942, Saudi Arabia; dr_kmq@hotmail.com; 4 Oral and Maxillofacial Prosthodontics Department, Faculty of Dentistry, King AbdulAziz University, Jeddah 21589, Saudi Arabia; eaattar@kau.edu.sa; 5 Department of Prosthetic Dental Science, College of Dentistry, King Saud University, Research Chair for Bio-logical Research in Dental Health, Riyadh 11545, Saudi Arabia; fvohra@ksu.edu.sa

**Keywords:** work quality, laboratories, crowns, dental, technician, shade matching, adaptation

## Abstract

Dental laboratories (LABs) are integral to the performance of a dentist in providing successful oral rehabilitation. The aim of this study was to compare the adaptation, contour, contacts, and shade matching of different government and commercial dental LABs in the fabrication of porcelain fused to metal (PFM) crowns. Thirty-two dental LABs were selected to fabricate PFM crowns (one PFM crown each). Marginal adaptation, contour, proximal contacts, and shade matching were evaluated. Evaluation of the crowns’ quality was performed following modified USPHS/FDI criteria. Visual and colorimeter assessments were employed to evaluate shade matching. Differences between groups were examined by Pearson’s Chi-square and Fisher’s exact test. The quality of marginal adaptation of crowns was good in 81.25%, however the quality of contours, contacts, and shade matching was compromised in 43.75%, 59.38%, and 39% of all LABs, respectively. Visual and colorimeter shade matching was acceptable in 62.5% and 80% of LABs in the cervical third and middle third regions of crowns, respectively, however in the incisal third the shade matching was unacceptable in nearly 60% of LABs. Commercial laboratories showed significantly better contours and shade matching, but not marginal adaptation. However, no significant differences were found in comparison of proximal contacts between the groups.

## 1. Introduction

Oral rehabilitation commonly involves provision of dental prosthesis fitted by dentists and fabricated by laboratory (LAB) technicians. However, nearly 4% of all the restorations made in laboratories are returned by clinicians for remaking [1]. The quality of the prostheses generated by a dental laboratory is greatly dependent on the knowledge and experience of both parties, their attention to detail, and the equipment used [2,3,4,5]. Individual possible factors which may render a crown unacceptable include poor impressions, lack of occlusal and axial reduction, poor mounting, inaccurate inter-occlusal record, and poor laboratory work. The successful rehabilitation of teeth by means of prosthetic devices requires meticulous teamwork and communication between the dentist and the dental laboratory technicians. Therefore, a clear prescription from the clinician can prevent unnecessary remakes, delays, and/or pre-insertion adjustments [6]. Although it is possible to reproduce the natural characteristics of dental tissue with dental porcelain, esthetic excellence, particularly the color matching of a porcelain restoration to natural dentition or existing restoration, is a challenging task [7,8].

An acceptable and clinically successful restoration must display adequate fit and adaptation, shape and contours, esthetic appearance with shade matching, and suitable hygienic contacts. Poor fit and adaptation lead to micro leakage and secondary caries, however shape and contour errors cause asymmetry and plaque accumulation [9,10,11]. Fabrication of acceptable esthetic restoration requires the integration of several critical factors, including an individual’s perception of color and the environment during visual shade selection, knowledge of dental anatomy, compatibility with materials, preparation designs, and accurate impressions [12,13]. In dentistry, porcelain fused to metal (PFM) crowns are the most commonly indicated in oral rehabilitations, however the challenge for shade matching in PFM crowns is high due to the metal substructure [14]. In addition, the metal copings for PFM restorations are fabricated with variable techniques, including casting, subtractive manufacturing or milling and additive manufacturing or 3-D printing. Furthermore, the opaque metal substructure is veneered with conventional and contemporary ceramic technologies, such as sintering, computer-aided design, computer-aided manufacturing, and heat pressing [11]. Therefore, the different complex fabrication procedures make PFM restorations particularly prone to color and fit inaccuracies. It is suggested that the color differences within and among commercially available dental ceramics may contribute to errors in the shade duplication process [15,16,17]. However, continued research has incorporated virtual technology and digital color measurement systems into clinical dentistry, to avoid the variability of conventional visual shade selection procedures [18]. Among the contemporary systems for shade matching, ShadeEye-NCC is a device that provides intraoral readings with four porcelain systems (Vita 3D-Master, Vitapan Classical, Vintage Halo, and Biodent) in clinical use and along with CIE L*a*b* coordinates. The system allows registration of direct color coordinate specifications without the use of mathematic manipulation. The system is designed to measure the shades of natural teeth or restorations through a disposable flat contact tip measuring 2.5 mm in diameter [19].

A dentist prepares teeth and the impression of the tooth designated for restoration. It is primarily the technician’s skills and judgment that are responsible for the production of a clinically acceptable restoration. Therefore, the knowledge and skills of the laboratory technician are highly critical in producing a successful restoration [5,6]. In a recent study by Jathmi et al., 70% of cases fabricated by LAB technicians were able to duplicate the shade accurately as determined by spectrophotometer [20]. In a similar study by Alshiddi et al. variations in the accuracy of indirect restorations made by laboratories managed by dental schools and those privately managed were assessed [21]. They reported wide variations in the accuracy and standards of restorations, and overall found no significant difference in restorative quality among privately and state-run LABs. However they failed to report the esthetic, contours, and contact parameters critical for the success of PFM restorations. To our knowledge from indexed literature, there are few research reports showing the accuracy of dental laboratories for fitting, contouring, contacting, and shade matching of dental crowns. It is hypothesized that there is no difference among laboratories (government and commercial) in the fabrication of high-quality and shade-matched PFM crowns. Therefore, the aim of this study was to assess and compare the accuracy of different dental laboratories (commercial and government) in the fabrication of high-quality and esthetic porcelain fused to metal (PFM) restorations.

## 2. Materials and Methods

**Figure 1:**
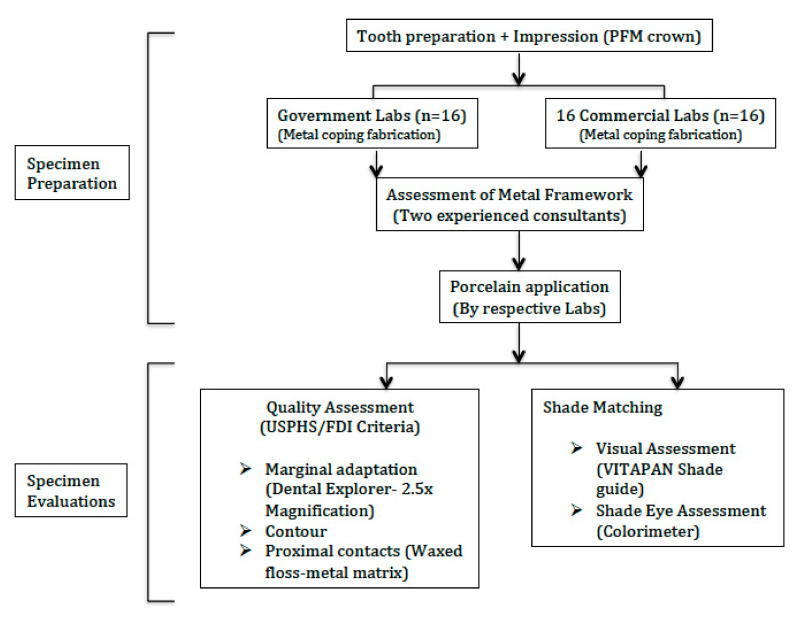
Flow diagram of the study methods.

In this in vitro cross-sectional study, 32 dental laboratories (16 government and 16 commercial) were included. The LABs were randomly selected but equally divided into commercial and government. Government LABs were from health sectors, as well as dental school-based laboratories. The study was performed at the College of Dentistry, King Saud University, from Jan 2019 to May 2019. Figure 1 presents the processes performed in the study as a flow diagram.

### 2.1. Specimen Preparation

Tooth preparation for a PFM crown on a maxillary left central incisor (Ivorine tooth #21), mounted on a dentoform jaw model (NISSIN Dental Products Inc., Kyoto, Japan), was performed using an index having reductions of 1.5 mm facial (2 plane), 1.0 mm palatal, and 2.0 mm incisal, with facial shoulder margins and palatal chamfer margins. The prepared tooth #21 in the dentoform jaw model was then unscrewed and fitted into the socket-like hole for tooth #21 on a study cast model for a simulated patient. The prepared Ivorine tooth was secured on the study cast. Thirty-two final impressions, in a light- and heavy-body polyvinyl siloxane impression material (Aquasil, Dentsply, York, PA, USA), in plastic full-arch trays were taken for the master cast by a senior clinician (MSB). Quality of the impressions was carefully inspected by means of a 2.5× magnification loupe. Final impressions were sent to the laboratories on the same day as they were recorded, along with the opposing casts and bite records (Occlufast, Zhermack, Italy). A standard work authorization form was prepared, including impression pouring into a die stone for fabrication of a working cast model, pindexing, die trimming, waxing, and casting of the metal substructure with a thickness of 0.3–0.4 mm. These steps of the sample preparation were performed by experienced restorative consultants (MBS and YAF). Metal frameworks were assessed by means of a sharp-end stainless steel measuring gauge (Patterson Iwanson Spring Caliper, Patterson Dental, St. Paul, MN, USA), by an experienced single operator (YAF). Metal frameworks were adjusted to meet the standard thickness (if needed) and returned to the laboratories for porcelain application with shade-mapping (A3.5 cervical 3rd, A2 middle 3rd, and A1 incisal 3rd). The crowns in all labs were fabricated by a representative trained and qualified technician with a minimum of 5 years of professional experience. The final crowns were collected from all laboratories and a code was randomly assigned to each case.

### 2.2. Quality Evaluation

**Figure 2:**
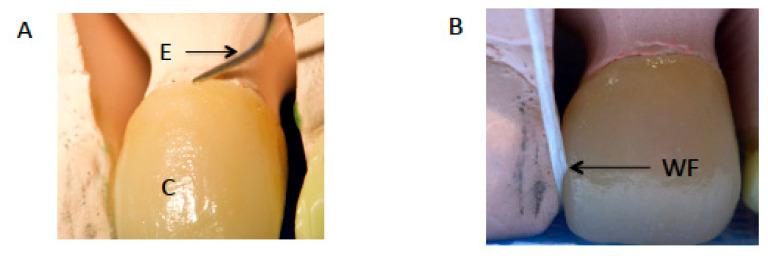
(**A**) Marginal accuracy assessment of crown (C) with sharp explorer (E). (**B**) Assessment of contours and contacts with waxed floss (WF).

Two experienced clinical restorative consultants (with 10 years of experience, MBS and YAF) using 2.5× magnification loupes evaluated the quality and shade of each crown anonymously. In the few cases where the examiners assessment was different, a third examiner assessment was performed and the majority decision was adopted (Figure 2A,B). Inter-examiner reliability was assessed with a kappa score of 0.90.

A sharp new dental explorer (Hu-Friedy Mfg. Co., LLC, Chicago, IL, USA) was used for evaluation of the marginal adaptation. For evaluation of the proximal contacts, waxed dental floss (Oral-B, Procter & Gamble, Cincinnati, OH, USA) and three different thicknesses (25, 50, and 100 µm) of Shim stock aluminum foil (Shim-in-A-Can, DMR, Oakville, ON, Canada) were used. Marginal adaptation, contour, and mesial and distal contact evaluation criteria were performed following the modified United States Public Health Service (USPHS) [22,23] World Dental Federation (FDI) [24] criteria as follows:

#### 2.2.1. Marginal Adaptation

R—Excellent/Ideal: Explorer does not catch; continuous adaptation and indistinguishable margins.

S—Acceptable: Explorer detects but cannot penetrate the marginal area.

T—Acceptable with modifications: Detectable and slightly overextended margins.

V—Unacceptable: Gross marginal discrepancies upon explorer examination. Remake is necessary.

#### 2.2.2. Contour

R—Excellent/Ideal: Contour follows normal physiologic tooth contour with no adjustments needed.

S—Acceptable: Slightly under- or over-contoured; no modifications needed.

T—Acceptable with modifications: Over-contoured restoration requires re-contouring.

V—Unacceptable: Gross under- or over-contoured crown. Remake is necessary.

#### 2.2.3. Proximal Contacts

R—Excellent/Ideal: Dental floss can be inserted under pressure and/or 25 µm (but not 50 µm) metal matrix can be inserted.

S—Acceptable (light): Dental floss can be inserted with minimal resistance and/or 50 µm (but not 100 µm) metal matrix can be inserted.

T—Acceptable with modifications (too strong): Dental floss can be inserted only with pressure and force and/or 25 µm metal matrix cannot be inserted. Crown needs adjustment before cementation.

V—Not acceptable (open): Dental floss passes through without any resistance, and/or 100 µm metal matrix can be inserted. Crown needs redo.

Readings of the marginal integrity, contour, and mesial and distal contacts were collected and analyzed, and data were analyzed.

A single PFM crown from each laboratory (commercial and government) was evaluated. For marginal assessment, crown contour and proximal contacts, 32, 32, and 64 evaluations in duplicate (two examiners) were performed.

### 2.3. Shade Evaluation

Both visual assessment and digital colorimeter shade evaluations were conducted. The visual shade evaluation included the VITA PAN classic shade guide (H. Rauter GmbH & Co. Kg, Homberg, Germany). Each clinician evaluated the shade separately (twice), with a 2-week interval between the evaluations. All visual examinations were conducted in the same dental office between 11:00 a.m. and 3:00 p.m by restorative dentistry consultants (MBS and YAF). The shade on which both clinicians agreed was chosen for the cervical, middle, and incisal areas of the crown. Colorimeter readings were performed by means of the ShadeEye-NCC Colorimeter (Shofu Inc., Kyoto, Japan) twice at a 2-week interval. In case of discrepancy in the two readings, a third evaluation was conducted (TJ). A total of 64 visual and 128 colorimeter assessments were performed in total for shade evaluations.

### 2.4. Statistical Analysis

The quantitative data was analyzed using the Statistical Package for the Social Sciences (SPSS, v. 20 for Windows, IBM Statistics Inc., Chicago, IL, USA). Descriptive analysis was performed to present an overview of the findings. Two-way cross-tabulation was used for comparison of proportions for each parameter between government and commercial LABs. Differences between groups were examined by Pearson’s Chi-square test and Fisher’s exact test for linear trends. A Bonferroni correction was performed due to repeated measurements and a *p* value of 0.05/8 = 0.0062 was considered as statistically significant.

## 3. Results

### 3.1. Quality

#### 3.1.1. Marginal Adaptation

A majority of the laboratory (n = 22, 68.8%) fabricated crowns showed acceptable marginal adaptation, including 45.5% government and 54.5% commercial LABs (Table 1). Four commercial LABs only showed excellent margin and adaptation. By contrast, two and four government LABs presented unacceptable and acceptable with modification and marginal adaptation, respectively (Table 1). Commercial LABs exhibited comparable marginal adaptation compared to government LABs (*p* > 0.006). Overall, 80% of crowns fabricated by LABs presented excellent or acceptable margin adaptation, with 20% either requiring modifications or were unacceptable.

#### 3.1.2. Crown Contours

Twelve laboratory (37.5%) fabricated crowns showed excellent contours, including 83.3% commercial LABs and 16.6% government LABs (Table 1). Regarding acceptable crowns (6 (18.7%)), 66.6% were from commercial LABs and 33.3% were made in government LABs. The acceptable with modifications and unacceptable crowns, fabricated by government LABs, were 8 (80%) and 4 (100%), respectively. By contrast, only 2 and 0 crowns in the acceptable with modifications and unacceptable category were made in commercial LABs. Commercial laboratory fabricated crowns showed significantly better contours than government LAB fabricated crowns (*p* = 0.002). Overall, 14 (43%) LABs made crown contours which either required modifications or were unacceptable (Table 1).

#### 3.1.3. Proximal Contacts

Only 25% (n = 8) of all crowns had excellent mesial contacts, including 6 from commercial LABs and 2 from government LABs (Table 2). A majority of LABs (62.5%, n = 20) produced crowns acceptable with modifications of mesial contacts, equally distributed between government (n = 10, 50%) and commercial (n = 10, 50%) LABs respectively. Overall, 6.2% of crowns fabricated had unacceptable contacts, and there was no statistical difference in mesial proximal contacts fabricated by commercial and government LABs (*p* = 0.125).

Ten laboratory (31.3%) fabricated crowns showed excellent distal contacts, including 80% commercial and 20% government LABs (Table 2). Half (n = 16) of all LAB fabricated crowns had distal contact which was either unacceptable or acceptable with modifications. A total of 75% (n = 6) of the crowns with unacceptable distal contacts were fabricated by government LABs, however only two commercial LABs showed unacceptable distal contacts. Commercial laboratories fabricated crowns with better distal proximal contacts; however, no statistically significant difference was found between the two LAB groups (*p* = 0.106).

### 3.2. Shade Matching

#### 3.2.1. Visual Assessment

Overall, 62.5% (n = 20) of the LABs showed shade matching in the cervical third. A total of 75% (n = 12) of the commercial LABs and 50% (n = 8) of the government LABs produced matching shades for cervical thirds of their crowns. However, no statistically significant difference was found between the two LAB groups (*p* = 0.273). For the middle third, 75% (n = 24) of the LABs showed shade matching (Table 3). An equal proportion (n = 12) of commercial LABs and government LABs showed successful shade matching. Therefore, no statistically significant difference was found between the tested groups in matching the middle third (*p* = 1.0). In the incisal third, only 43.7% (n = 14) of the LABs showed shade matching (Table 3). Furthermore, 68.7% (n = 11) of commercial LABs and only 18.7% (n = 3) of government LABs succeeded in matching the incisal third shade. Fisher’s exact test showed a statistically significant difference between government and commercial LABs in the incisal third (*p* = 0.001).

The number of crown thirds that were successfully matched by each LAB category is shown in Table 3. Nearly 44% (n = 7) of the commercial LABs and 6.2% (n = 1) of government LABs perfectly matched all three thirds. Overall, commercial LABs produced PFM crowns with significantly better visual shade matching than government LABs (*p* = 0.004).

#### 3.2.2. ShadeEye Assessment

Table 4 presents the ShadeEye color matching outcomes among the LAB groups. We found that 62.5% of all LABs produced crowns with matching shades, including 8 (40%) government LABs and 12 (60%) commercial LABs. A total of 50% (n = 8) of the government LABs and 75% (n = 12) of the commercial LABs matched the cervical third in their crowns. Furthermore, 50% of government LABs and 25% of commercial LABs failed to match crown shade. Fisher’s exact test showed no statistically significant difference between government and commercial LABs in the cervical third (*p* = 0.273). In the middle third, 84% of crowns were shade matched, including 51% government and 48% commercial LABs. About 81% (n = 13) of commercial LABs and 87.5% (n = 14) of government LABs showed shade matching in the middle third (Table 4). Statistically, both LAB types showed similar accuracy of shade matching in the middle third (*p* = 1.0).

A mere 37.5% of LABs showed shade matching in the incisal third, including 10 (83%) commercial and only 2 (16.6%) government LABs. A staggering 87.5% (n = 14) of government LABs failed to match shades in the incisal third. A total of 62.5% (n = 10) of commercial LABs were able to produce PFM crowns with accuracy of color. The overall ability of government LABs to produce color-matching crowns was significantly lower than commercial LABs (*p* = 0.001).

## 4. Discussion

This comparative study aimed to evaluate the performance of different prosthetic LABs in the production of high-quality and esthetic PFM crowns. It was based on the hypothesis that there is no significant difference among laboratories (government and commercial) in marginal adaptation, contours, contacts, and shade matching of PFM crowns. It was observed that contours and color matching of PFM crowns was significantly different (*p* < 0.006), and government LABs showed poor outcomes compared to commercial LABs. Therefore the hypothesis was rejected. A myriad of explanations are presented for the observed findings, including study methodology, ceramic materials, work environment, technical skills, and quality management.

PFM crowns are the most commonly used prosthetic restoration in dentistry [14]. For a successful PFM crown to be produced, careful diagnosis and clinical management of the case, as well as the choice of a good laboratory, are important factors. Dental laboratories differ in their abilities to acquire good equipment with high-quality materials, or to hire skillful technicians, which may affect the prosthesis from these LABs. In the present study, evaluations of quality were performed according to modified USPHS/FDI criteria. The original USPHS criteria did not include proximal contact assessment [22,23]. Therefore, the FDI criteria were also included for the evaluation of interproximal contacts. Previous studies have validated the application of these criteria for chair-side reproducible assessments of direct and indirect dental restorations [25,26,27]. In addition, to evaluate marginal integrity, a sharp dental explorer is considered to be a reliable clinical tool for the detection of marginal gaps [28,29]. Moreover, studies have used dental floss for the evaluation of proximal contacts for both direct and indirect restorations [30,31]. However, it is recommended to use metal strips with specific thicknesses for more reliable results [23,32,33,34]. In the present study, both methods were used and integrated in the evaluation criteria, and the relationship of the tinfoil thickness to the resistance of dental floss was tested and verified in all cases. The use of a master cast to evaluate the crowns with adjacent teeth that are fixed and immobile, in contrast to the natural teeth that were examined in the other studies, can also explain these results [32,33,34].

In this study, commercial laboratories performed better than government laboratories in the fabrication of PFM crowns (contours and shade matching). Although the percentage of unacceptable PFM crowns from both types of laboratories was low, a difference between the two laboratory types was not present. Previous studies have reported similar outcomes [21]. In the study by Alshiddi et al., margins of the crowns fabricated by commercial LABs were not statistically different to the crowns made by dental school laboratories [34]. This is in line with the findings of the present study.

Interestingly, PFM crown shades were assessed visually and digitally (by means of a colorimeter), since these two methods are considered to be the most commonly used tooth color measuring techniques [35]. In addition, Vita TM classic has been documented as the most commonly used shade guide [36]. Although the traditional clinical method used for evaluating shades is a visual comparison of the tooth or the restoration with commercial shade guides [37], possible limitations, including the subjectivity of color perception between and among individuals, eye fatigue and color deficiencies [37], mandated the use of an adjunct method to evaluate crown shades. In the present study, three sites were measured in each crown; therefore, ShadeEye-NCC was used as the adjunct measuring tool, having a smaller terminal measuring tip with a diameter of 2.5 mm. It has been documented that visual shade confirmation is recommended when the ShadeEye-NCC colorimeter is used [38].

In the present study, visual shade assessments exhibited better performance of all LABs in the middle thirds, followed by cervical thirds. The worst shade matching results were reported in the incisal thirds. Although commercial LABs performed better than or equal to government LABs in all thirds, differences among the two lab groups were not significant in cervical and middle thirds. In the incisal third, however, the results were significantly different between the tested groups. These results were confirmed by the colorimeter readings; however, for the middle thirds, 14 government LABs matched the shade compared with 13 commercial LABs. The inferior performance of the LABs, especially in matching the incisal thirds, is attributed to the translucency and thickness of porcelain in this area, the variations in the veneering ceramics, the difference in illumination in LABs, experience of LAB technicians, and the work ethics in different LABs. Findings for the visual and colorimeter readings in this study were identical in the cervical thirds. However, for the middle and incisal thirds, small differences between the two methods were reported. Since ShadeEye-NCC is designed with a flat detecting tip, for the measurement of surfaces, it is prone to edge-loss effects, which may lead to incorrect readings. These findings were also reported by Yuan et al. [38]. For accurate matching of crown shades in all thirds, both test methods confirmed the significant superior performance of commercial LABs compared with government LABs.

The outcomes of the study should be interpreted in light of the possible limitations. Only one representative specimen from each LAB was used for evaluations and this could result in under- or over-expressing the disparity among LABs. In addition, dental LABs produce multiple types of prostheses including orthodontic appliances, crowns, fixed partial dentures, removable dentures, trays, stents, and maxillofacial prostheses. However, the present study assessed a simple and most commonly fabricated prosthesis (PFM crown) to represent the work of LABs. These simplifications due to methodological restrictions suggest that the quality and accuracy findings should not be applicable to other LAB products and are limited to PFM crowns. The outcomes of this study highlight the disparity of prosthesis quality fabricated by different technicians and LABs. In addition, as outcomes of dental restorative treatments are directly dependent on restoration quality, it is critical to comprehend that improving the quality of LABs and technician skills will improve the quality of dental care in the community. This inconsistency in quality among LABs is attributed to the experience, training and skill of technicians, management of quality control, and working environment at the LABs. It is also observed that technicians are working longer hours in a challenging, performance- and incentive-based setting in commercial LABs, referred to as perform or perish. However, government setting, although allowing for better training and skill development, may be less challenging and the performance is not incentive based. As factors influencing the variation in outcomes of LABs are not investigated, further studies are recommended in this regard. Moreover, to increase the quality of dental health among the population, in line with the established standards, updating the knowledge and skills of technicians should be prioritized and a robust quality assessment and feedback process implemented.

## 5. Conclusions

Overall, the proximal contacts and marginal adaptation of PFM crowns fabricated by commercial and government managed LABs were comparable. However, contours and color matching of PFM crowns produced by commercial LABs were significantly better compared to government LABs.

## Figures and Tables

**Table 1:** Comparison of crown marginal adaptation and crown contour quality among different laboratories.

Marginal Adaptation				
Quality of Fit Criteria-Based Evaluation.	Government LABs (N and Row %)	Commercial LABs (N and Row %)	Total (N and Column %)	*p* Value
Excellent	0 (0)	4 (100)	4 (12.5)	0.007
Acceptable	10 (45.5)	12 (54.5)	22 (68.8)	
Acceptable with modifications	4 (100)	0 (0)	4 (12.5)	
Unacceptable	2 (100)	0 (0)	2 (6.20)	
**Crown Contours**				
Quality of Contours Criteria-Based Evaluation.	Government LABs (N and Row Percentage)	Commercial LABs (N and Row Percentage)	Total (N and Column %)	*p* Value
Excellent	2 (16.6)	10 (83.4)	12 (37.5)	0.002
Acceptable	2 (33.4)	4 (66.6)	6 (18.75)	
Acceptable with modifications	8 (80)	2 (20)	10 (31.25)	
Unacceptable	4 (100)	0 (0)	4 (12.5)	

**Table 2:** Comparison of goodness of mesial and distal proximal contacts among different laboratories.

Mesial Proximal Contact				
Quality of Contacts Criteria-Based Evaluation	Government LABs (N and Row %)	Commercial LABs (N and Row %)	Total (N and Column %)	*p* Value
Excellent	2 (25)	6 (75)	8 (25)	0.125
Acceptable	2 (100)	0 (0)	2 (6.25)	
Acceptable with modifications	10 (50)	10 (50)	20 (62.5)	
Unacceptable	2 (100)	0 (0)	2 (6.25)	
**Distal Proximal Contact**				
Quality of Contacts Criteria-Based Evaluation	Government LABs (N and Row Percentage)	Commercial LABs (N and Row Percentage)	Total (N and Column %)	*p* Value
Excellent	2 (20)	8 (80)	10 (31.3)	0.106
Acceptable	4 (66.6)	2 (33.4)	6 (18.7)	
Acceptable with modifications	4 (50)	4 (50)	8 (25)	
Unacceptable	6 (75)	2 (25)	8 (25)	

**Table 3:** Comparison of visual shade matching overall and among different thirds of PFM (porcelain fused to metal) crowns fabricated by different laboratories.

Visual Shade Matching, Cervical 3rd				
Accuracy of Shade, Criteria-Based Evaluation.	Government LABs (N and Row %)	Commercial LABs (N and Row %)	Total (N and Column %)	*p* Value
Matching	8 (40)	12 (60)	20(62.5)	0.273
Non-Matching	8 (66.6)	4 (33.4)	12 (37.5)	
**Visual Shade Matching, Middle Third**				
Matching	12 (50)	12 (50)	24 (75)	1.0
Non-Matching	4 (50)	4 (50)	8 (25)	
**Visual Shade Matching, Incisal Thid**				
Matching	3 (21.5)	11 (78.5)	14 (43.7)	0.004
Non-Matching	13 (72.3)	5 (27.7)	18 (56.3)	
**Overall Visual Shade Matching (All Thirds)**				
Complete Matching	1 (12.5)	7 (87.5)	8 (25)	0.005
2/3rd matching	11 (61.2)	7 (38.8)	18 (56.25)	
1/3rd matching	0 (0)	2 (100)	2 (6.25)	
None matching	4 (100)	0 (0)	4 (12.5)	

**Table 4:** Comparison of ShadeEye color matching overall and among different thirds of PFM crowns fabricated by different laboratories.

ShadeEye Shade Matching, Cervical Third				
Accuracy of Shade, Criteria-Based Evaluation.	Government LABs (N and Row %)	Commercial LABs (N and Row %)	Total (N and Column %)	*p* Value
Matching	8 (40)	12 (60)	20(62.5)	0.27
Non-Matching	8 (66.6)	4 (33.3)	12 (37.5)	
**ShadeEye Shade Matching, Middle Third**				
Matching	14 (51.8)	13 (48.1)	27 (84.4)	1.0
Non-Matching	2 (40)	3 (60)	5 (15.6)	
**ShadeEye Shade Matching, Incisal Third**				
Matching	2 (16.6)	10 (83.3)	12 (37.5)	0.004
Non-Matching	14 (70)	6 (30)	20 (62.5)	
**Overall ShadeEye Shade Matching (All Thirds)**				
Complete Matching	0 (0)	8 (100)	8 (25)	0.001
2/3rd matching	10 (83.4)	2 (16.6)	12 (37.5)	
1/3rd matching	4 (40)	6 (60)	10 (31.3)	
None matching	2 (100)	0 (0)	2 (6.2)	

## Data Availability

The data presented in this study are available on request from the corresponding author.
